# Fear of Pain as a Predictor for Postoperative Pain Intensity among the Patients Undergoing Thoracoscopic Surgery

**DOI:** 10.1155/2022/2201501

**Published:** 2022-06-15

**Authors:** Yang Luo, Jingting He, Lihong Bao, Heng Meng, Cuihuan Hu, Quan Chen

**Affiliations:** ^1^Department of Thoracic Surgery, Union Hospital, Tongji Medical College, Huazhong University of Science and Technology, Wuhan, China; ^2^Cancer Center, Union Hospital, Tongji Medical College, Huazhong University of Science and Technology, Wuhan, China; ^3^School of Public Health, Huazhong University of Science and Technology, Wuhan, China; ^4^School of Nursing, Huazhong University of Science and Technology, Wuhan, China; ^5^Department of Radiology, Union Hospital, Tongji Medical College, Huazhong University of Science and Technology, Wuhan, China

## Abstract

**Background:**

Fear of pain (FOP) has been recognized as an influential moderator and determinant of the perception and disability of chronic pain. However, studies on FOP in postoperative acute pain are few and inconsistent.

**Objective:**

To explore whether FOP is related to pain intensity after thoracic surgery and provide a reference for FOP study in postoperative pain.

**Methods:**

From February to March 2022, 89 patients completed Chinese Version of Fear of Pain-9 Items (FOP-9), Chinese version of the Brief Pain Inventory (BPI, including least, worst, and average pain) and Simplified Chinese version of the Pain Catastrophizing Scale (PCS). Correlation analyses and mediation analyses were used for exploring the relationship between factors.

**Results:**

Mediation analyses showed that the total effects of FOP-9 on BPI all were significant (least pain: effect = 0.085, *p*=0.013, 95% CI = 0.019∼0.151; worst pain: effect = 0.116, *p*=0.004, 95% CI = 0.037∼0.196; average pain: effect = 0.102, *p*=0.005, 95% CI = 0.031∼0.174) indicating that FOP-9 was a predictor to BPI. The 95% bias-corrected bootstrap confidence interval of estimate of indirect effect between FOP-9 and least pain/average pain through PCS was −0.036∼0.024 and −0.003∼0.069 (all contain zero), which indicated that PCS is not a mediator between FOP-9 and least pain/average pain. However, the estimate of indirect effect between FOP-9 and worst pain through PCS were 0.048 (95% CI = 0.095∼0.088), and direct effect was not statistically significant (95% CI = −0.017∼0.153), indicating that PCS acted as a complete intermediary between FOP-9 and worst pain. FOP-9 and PCS showed significant positive prediction effect on worst pain.

**Conclusions:**

Both trait FOP and state FOP were associated with higher postoperative pain reports after thoracic surgery. Trait FOP influences postoperative pain through the mediating effect of state FOP.

## 1. Background

Every one of us may experience different types and levels of pain triggered by various situations, which happens often in many different stages of our lives. Pain is subjective and affected by many aspects such as psychosocial factors, which means that people can report and feel different pain sensations for the same injury. These sensations may be directly associated or determined by a person's different psychological features, which makes our brain a major actor in pain perception, since it is responsible to process the feelings that run through our bodies after painful events [1]. Painful memories of past events lead to a type of personality traits called fear of pain (FOP) [2], that can be described as the verbal, physiological and overt behavior responses to potential or anticipated painful experiences [3].

Postoperative pain, a type of acute pain, is still the most common complication of thoracoscopic surgery. Although the pain caused by this invasive procedure is classified as less severe than thoracotomy [4], it can cause a variety of adverse effects on the body, e.g., increasing oxygen consumption and pulmonary complications, which limit physical activities, increase family burden, and may even develop into chronic pain [5].

At present, FOP has been recognized as an influential moderator and determinant of the perception and disabilities caused by chronic pain [6–8]. However, different than what has been already studied about the way the body respond to chronic pain, the scholarly conclusions on the role of FOP in acute pain are still inconsistent. Most studies point out that FOP plays a causal pathway between pain and physical disabilities [9–11], triggering a hypervigilant behavior to avoid other types of pain, especially in individuals suffering from acute pain. Differently, a few reports [12, 13] indicate that higher levels of FOP are not associated with pain intensity.

In addition, different forms of the persistent and stable FOP (trait FOP) in individuals suffering from chronic pain, the FOP in cases of acute pain is likely to develop as a series of temporary mental and physical reactions (state FOP) directly related to a given moment. In healthy adults, this trait FOP does not lead to attentional bias for pain-threatening words, whereas state FOP tends to put the individual in a mental state that is affected by this biased perception [14]. However, research on the relationship between trait FOP, state FOP, and pain perception are scarce, so more studies are still needed to understand how these mind states truly affect one's behavior.

The proposed study aims to explore whether FOP is related to pain intensity after thoracic surgery, and better understand the relationship between trait FOP, state FOP, and postoperative pain.

## 2. Material and Methods

### 2.1. Design and Setting

This study is a single-center, cross-sectional study of thoracic hospitalized patients at Wuhan Union Hospital (tertiary hospital) in Wuhan, China. The inclusion criteria were as follows: (1) patients over 18 years old, (2) waiting for thoracoscopic surgery, and (3) no presence or history of a neurological or psychiatric disorder. Furthermore, the exclusion criteria include (1) cancelled thoracoscopic surgery, (2) admitted to other departments after surgery, and (3) uncooperative behavior. All participants provided written and oral informed consent, and the research design was approved by the Institutional Ethics Board of Wuhan Union Hospital of Tongji Medical College, Huazhong University of Science and Technology (No. 20200351). Clinical trial registration was also properly conducted (No. ChiCTR2200056651). This study was supported by general program of Hubei Provincial Natural Science Foundation of China (No. 2021CFB588) and the funder did not play any role in the designing, conducting, or reporting of this study.

### 2.2. Outcomes

In the past studies, trait FOP was usually measured by a Fear of Pain Questionnaire [15]. In this study, the same methodology was adopted and we used the simplified Chinese version of the 9 items [16] (FOP-9) to obtain information on a patient's trait FOP at admission. FOP-9 is a 5-point ordinal Likert scale and the total score ranges from 9 to 45, with higher score meaning more severe FOP.

Subsequently, the Chinese version of the Brief Pain Inventory [17] (BPI) was used to collect data on postoperative pain intensity and emotional responses. To reduce the amount of time needed and increase patient compliance, only items 3 (worst pain), 4 (least pain), and 5 (average pain) of the BPI were asked every morning from the first to third postoperative days.

Additionally, the simplified Chinese version of the Pain Catastrophizing Scale [18] (PCS) was completed to screen the patients' state FOP on the third day after surgery. PCS is a 5-point instrument that yields 3 subscale scores assessing rumination, magnification, and helplessness.

### 2.3. Potential Prognostic Factors

The selection of potential prognostic factors was based on systematic reviews. Data on age, gender, body mass index (BMI), educational background, level of current pain, psychological pain (measured by the psychological pain thermometer method [19]), depression (measured by Patient Health Questionnaire-9, PHQ-9) [20], operation method, and use of analgesic drugs were collected by two trained researchers. All patients are given a constant basic dosage of analgesic drugs that could be increased or decreased according to the patient's report of pain levels.

### 2.4. Statistics

The statistical analyses were performed in SPSS version 25.0 for Windows (Chicago, IL, USA). All participants that agreed on providing data were included. Descriptive data were presented as counts and percentages for categorical data and means and standard deviations or medians and interquartile ranges were used to examine continuous normally distributed data and data with skewed distribution. The correlations of study variables were analyzed by correlation analyses. Model 4 of PROCESS [21] macro for SPSS was used for exploring the mediation effect. The bias-corrected confidence interval (CI) was calculated with 5000 bootstrapping resamples. The level of the CIs was set at 95% and that of the tests at 5%. All *p* values were two-sided.

## 3. Results

**Figure 1:**
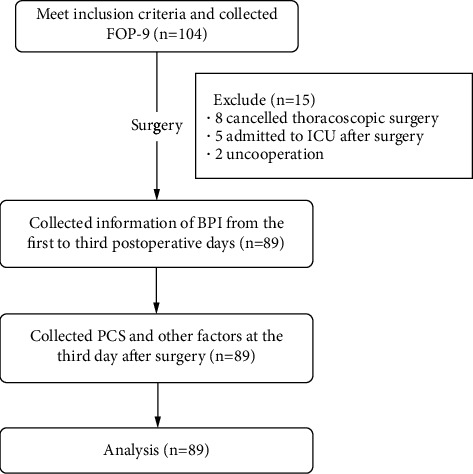
Included participants and progress of measures. FOP-9, Fear of Pain-9 Items; PCS, Pain Catastrophizing Scale; ICU, Intensive Care Unit.

From February to March 2022, 104 patients met the inclusion criteria and 89 patients were enrolled. Details of the recruited patients are presented in Figure 1 and baseline characteristics of the study population are listed in Table 1. The collected BPI data shows that 98.9% (1/89) of patients report worst pain of 1 point or more, and 43.8% reported an average pain score of 4 or more.

Table 2 provides the correlations between the variables studied. The results indicated that lower levels of pain were positively related to the use of analgesic drugs (*r* = 0.240, *p* < 0.05). Furthermore, patients who reported higher pain levels were more likely to ask for analgesics. Severe pain and average pain were associated with FOP-9 (*r* = 0.257, *p* < 0.05 and *r* = 0.292, *p* < 0.01, respectively), PCS (*r* = 0.374, *p* < 0.01 and *r* = 0.317, *p* < 0.01, respectively), and the use of analgesic drugs (*r* = 0.459, *p* < 0.01 and *r* = 0.456, *p* < 0.01, respectively). The coding of variables can be seen in details in Table 1.

As shown in Table 3, the results of mediation analyses pointed out that the total effects of FOP-9 on BPI were significant (low pain: effect = 0.085, *p*=0.013, 95% CI = 0.019∼0.151; worst pain: effect = 0.116, *p*=0.004, 95% CI = 0.037∼0.196; average pain: effect  = 0.102, *p*=0.005, 95% CI = 0.031∼0.174) indicating that FOP-9 was a predictor to BPI.

The 95% bias-corrected bootstrap confidence interval of estimate of the indirect effect between FOP-9 and least/average pain through PCS was −0.036∼0.024 and −0.003∼0.069 (all contain zero), indicating that PCS is not a mediator between FOP-9 and least pain/average pain. However, the estimate of indirect effect between FOP-9 and worst pain through PCS were 0.048 (95% CI = 0.095∼0.088), and direct effect was not considered statistically significant (95% CI = −0.017∼0.153), which indicates that PCS acted as a complete intermediary between FOP-9 and worst pain. FOP-9 and PCS were considered to have a significant positive prediction effect on patients who reported severe pain (Table 4).

## 4. Discussion

Surgical procedures are associated with different levels of pain, which are usually acute rather than chronic in nature [22]. Although patients that went through thoracoscopic surgery experienced less pain than those that underwent thoracotomy, 98.9% of patients included in this study still reported acute postoperative pain. Of these, 43.8% reported average pain of more than 4 points, which is generally considered to be sleep-affecting. These results suggest that the medical community must pay more attention to the way patients experience acute pain after surgical interventions.

The way the body experiences postoperative pain depends on different factors. In our study, patients hospitalized after thoracoscopic surgery had a median BPI score of 4 for severe pain and 3 for average pain. In patients undergoing thoracoscopic pulmonary surgery, we found that preoperative FOP, postoperative PCS, and postoperative use of painkillers were directly associated with postoperative pain intensity (Table 2). Interestingly, there was a positive correlation between the use of painkillers and pain intensity. We consider that this correlation might be due to the increased use of painkillers by patients who reported severe levels of pain.

Furthermore, FOP increased the severity of pain perceived by postoperative patients. A study reported that almost all patients had a fear of moderate to severe postoperative pain while in the preoperative period, and reduced FOP in the preoperative period may decrease postoperative pain and yield favorable patient outcomes [23]. It has been previously suggested in a similar study that preoperative fear of pain can predict the occurrence of short-term pain and disabilities after lumbar disc surgery [24]. A study conducted by Wang et al. [25] showed that painless subjects with high FOP had higher gray matter volumes in brain regions involved with pain sensations and fear processing, so they were more likely to perceive pain and fear. Furthermore, some laboratory studies have shown that FOP can predict pain perception and the rate of acute pain resolution in painless samples [12, 26, 27]. The outcomes of our study are aligned with these aforementioned conclusions. Our correlation (Table 2) and regression analyses (Table 4) showed that postoperative pain intensity was positively correlated with FOP-9 and PCS, indicating that patients with higher FOP would report higher pain intensity.

Our data also confirmed that trait FOP affects postoperative pain through state FOP. At present, only a few studies explored the effect of trait FOP on pain intensity, which makes our results a novelty in this field of study. Babel [2] previously reported that pain intensity can be predicted by state anxiety and trait pain anxiety. Similarly, Zheng et al. [27] conducted a clinical study that showed that trait FOP increases sensitivity to pain cues. However, despite these important conclusions on our studied phenomenon, the association between trait FOP and state FOP has not effectively explained yet. Results of our mediation effect analysis showed that trait FOP influences postoperative pain through state FOP. Moreover, data acquired from the correlation analysis also showed that the correlation coefficient of postoperative state FOP associated with pain intensity is higher. This means that state FOP in clinical practice requires more attention and targeted intervention measures should be provided for patients with high FOP. The mediating effect was not significant in patients who reported weak or average pain levels, which may be due to a great number of painless patients after surgery, resulting in extremely skewed data. Another reason may be that trait FOP is more likely to cause patients to become alert to higher pain levels, which leads to stronger state FOP and more reports of severe pain.

## 5. Limitations

Some of the patients included in this study reduced the use of analgesics not because of their levels of pain but because of some side effects cause by opioid analgesics, e.g., nausea, vomiting, and dizziness. In such cases, to reduce the patient's levels of pain, opioids were often replaced with nonopioid drugs. Therefore, the quantification of analgesic drugs in this study is inaccurate.

## 6. Conclusion

Both trait FOP and state FOP were associated with higher postoperative pain reports after thoracic surgery. Our data showed that trait FOP influences postoperative pain through the mediating effect of state FOP. These outcomes suggests that postoperative pain should be evaluated not only considering the use of drugs but also carefully considering the influence of psychological factors on the patient's levels of pain.

## Figures and Tables

**Table 1:** Baseline characteristics and categorization in analysis (*n* = 89).

Factors	Value	Range	Categorization
Age (years), median (IQR)	54 (49–61.50)	19–78	Original
Gender, freq (%)			Ordinal
Male	43 (48.3)		1
Female	46 (51.7)		0
BMI, mean(SD)	23.10 (2.93)	16.89–31.60	Original
Education, freq (%)			Ordinal
Primary school	7 (7.9)		1
Middle school	22 (24.7)		2
High school	27 (30.3)		3
University	33 (37.1)		4
Present pain, freq (%)			Original
Yes	10 (11.2)		1
No	79 (88.8)		0
FOP, median (IQR)	18 (14–22)	9–35	
Psychological pain, median (IQR)	0 (0–1)	0–8	Original
PHQ-9, median (IQR)	1 (0–2)	0–17	Original
BPI, median (IQR)			Original
Least	1 (0–3)	0–10	
Worst	4 (3–7)	0–10	
Average	3 (2–4)	0–10	
PCS, median (IQR)	30 (19.75–38)	9–52	Original
Operation method, freq (%)			Ordinal
Mediastinal	15 (16.9)		1
Wedge resection of lung	16 (18.0)		2
Segmental resection of lung	20 (22.5)		3
Lobectomy	38 (42.7)		4
Analgesic drugs, freq (%)			Ordinal
Decreased	14 (15.7)		1
Unchanged	53 (59.6)		2
Increased	22 (24.7)		3

IQR, interquartile range; freq, frequency; BMI, body mass index; SD, standard deviation; FOP, Fear of Pain-9 Items; PHQ-9, Patient Health Questionnaire-9; PCS, Pain Catastrophizing Scale.

**Table 2:** Correlation analysis (*n* = 89).

Variables	Correlation coefficient		
	Least pain	Worst pain	Average pain
Age^#^	−0.187	−0.037	−0.085
Gender^$^	0.014	−0.044	−0.065
BMI^#^	0.115	0.022	−0.029
Education^#^	0.064	0.104	0.042
Present pain^$^	−0.008	−0.107	−0.062
FOP-9^#^	0.125	0.257^*∗*^	0.292^*∗∗*^
Psychological pain^#^	0.157	0.146	0.055
PHQ-9^#^	−0.023	0.070	0.076
PCS^#^	0.092	0.374^*∗∗*^	0.317^*∗∗*^
Operation method^#^	−0.091	0.090	0.069
Analgesic drugs^#^	0.240^*∗*^	0.459^*∗∗*^	0.456^*∗∗*^

^#^Spearman; ^$^Kendall; FOP-9, Fear of Pain-9 Items; PHQ-9, Patient Health Questionnaire-9; PCS, Pain Catastrophizing Scale; ^*∗*^*p* < 0.05; ^*∗∗*^*p* < 0.01.

**Table 3:** Total effect, direct effect, and mediating (indirect) effect (*n* = 89).

Dependent variable		Effect	*t*	*p* value	95% CI
Least pain	Total	0.085	2.549	0.013	0.019∼0.151
	Direct	0.088	2.370	0.020	0.014∼0.162
	Indirect (PCS)	−0.003			−0.036∼0.024

Worst pain	Total	0.116	2.922	0.004	0.037∼0.196
	Direct	0.068	1.590	0.119	−0.017∼0.153
	Indirect (PCS)	0.048			0.095∼0.088

Average pain	Total	0.102	2.853	0.005	0.031∼0.174
	Direct	0.068	1.732	0.087	−0.010∼0.146
	Indirect (PCS)	0.034			−0.003∼0.069

FOP-9, Fear of Pain-9 Items; PCS, Pain Catastrophizing Scale; CI, confidence interval. FOP-9 as an independent variable, PCS as a mediating variable, and analgesic drugs as a covariate value.

**Table 4:** Regression tested by the mediation model in worst pain (*n* = 89).

Regression equation	PCS		Worst pain		Worst pain	
	*t*	95% CI	*t*	95% CI	*t*	95% CI
(Constant)	1.834	−0.755∼18.706	−1.263	−3.164∼0.705	−0.753	−2.702∼1.217
FOP-9	4.491^*∗∗*^	0.495∼1.282	1.590	−0.171∼0.153	2.922	0.372∼0.196
Analgesic drugs	1.033	−1.655∼5.238	4.694^*∗∗*^	0.920∼2.273	4.853^*∗∗*^	1.000∼2.388
PCS			2.581^*∗*^	0.013∼0.096		
*R*-squared	0.208		0.345		0.294	
*F*	11.299^*∗∗*^		14.945^*∗∗*^		17.908^*∗∗*^	

PCS, Pain Catastrophizing Scale; CI, confidence interval; FOP-9, Fear of Pain-9 Items; ^*∗*^*p* < 0.05; ^*∗∗*^*p* < 0.01. FOP-9 as an independent variable, PCS as a mediating variable, and analgesic drugs as a covariate value.

## Data Availability

The ACCESS data used to support the findings of this study are available from the corresponding author upon request.
